# Transcriptome Profiling in Systems Vascular Medicine

**DOI:** 10.3389/fphar.2017.00563

**Published:** 2017-08-25

**Authors:** Suowen Xu

**Affiliations:** Department of Medicine, Aab Cardiovascular Research Institute, University of Rochester School of Medicine and Dentistry, Rochester NY, United States

**Keywords:** transcriptome, microarray, RNA-sequencing, vascular medicine, gene profiling, long non-coding RNA

## Abstract

In the post-genomic, big data era, our understanding of vascular diseases has been deepened by multiple state-of-the-art “–omics” approaches, including genomics, epigenomics, transcriptomics, proteomics, lipidomics and metabolomics. Genome-wide transcriptomic profiling, such as gene microarray and RNA-sequencing, emerges as powerful research tools in systems medicine and revolutionizes transcriptomic analysis of the pathological mechanisms and therapeutics of vascular diseases. In this article, I will highlight the workflow of transcriptomic profiling, outline basic bioinformatics analysis, and summarize recent gene profiling studies performed in vascular cells as well as in human and mice diseased samples. Further mining of these public repository datasets will shed new light on our understanding of the cellular basis of vascular diseases and offer novel potential targets for therapeutic intervention.

## Introduction

According to a recent disease statistic report released by American Heart Association (AHA), cardiovascular diseases (CVD) remain the leading cause of death in America (Benjamin et al., 2017). The treatment of CVD also imposed a huge economic burden on the healthcare system (Benjamin et al., 2017). Deep understanding of the mechanism of CVD is a valuable approach for devising effective novel cardiovascular therapeutics.

With increasing number of transcriptomic studies (including microarray and RNA-sequencing) performed in cultured cells as well as in experimental mice or patients with CVD, we now have the capability to understand the influence of therapeutic intervention or gene perturbation on CVD outcome at genome-wide levels which were inaccessible in the past. However, the value of these transcriptomic data was always underestimated since most of the deposited data are not released to public until manuscripts are published. Therefore, it is critical to make large-scale efforts to mine, validate, and integrate the underlying information streams arising from various transcriptomics studies (Musunuru et al., 2017). To meet the increasing need of precision medicine, AHA has recently established the Institute for Precision Cardiovascular Medicine^1^, offering a new category of data-mining grants focused on harmonizing and mining CVD-based data for cardiovascular therapeutics. Therefore, in this article, I will summarize the workflow of transcriptomic profiling, basic bioinformatics analysis, and those profiling studies performed in vascular cells as well as human and mice diseased samples, aiming to provide a direct resource gallery in systems vascular medicine. Obviously, further mining of these publicly available datasets will provide a useful resource for understanding the cellular basis of atherosclerotic vascular diseases.

## Overview of Transcriptomic Analysis

**FIGURE 1:**
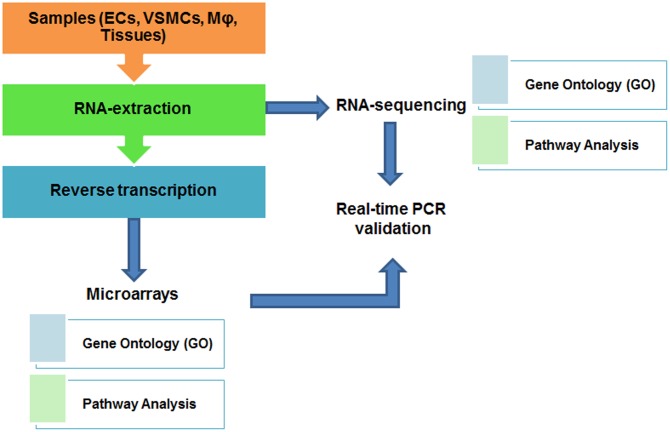
Workflow and downstream analysis of transcriptome studies.

For analyzing a small number of gene transcripts, quantitative real-time PCR or pathway-focused (such as pathways of angiogenesis or endothelial cell biology) gene expression analysis using PCR arrays (such as RT^2^ Profiler PCR Arrays from Qiagen) can be used. In order to understand genome-wide influence of different conditions on CVD outcome, DNA microarray and RNA-sequencing (RNA-seq) are frequently used. Traditional transcriptomic analysis was mostly performed by using DNA microarray, which employs dye (Cy3, Cy5) hybridization-based technology to analyze differential gene expression pattern under certain conditions (such as gene knockout, or drug/stimuli treatment), although microarray has several technical limitations (de Franciscis et al., 2016; Haase et al., 2016). Recently, with the advent of next-generation sequencing technology, transcriptomic analysis has transitioned to RNA-seq (Wang et al., 2009), to quantify the amount of transcripts including protein-coding genes (mRNA), splice variants, as well as long non-coding RNA transcripts (lncRNA) in biological samples at genome-wide level (Mortazavi et al., 2008). Comparatively speaking, RNA-seq has the capability to identify more differentially expressed genes in various cell types than gene microarray (Wang et al., 2009; Zhang et al., 2014). In addition, there are also some commercial lncRNA array services available, such as Arraystar LncRNA Expression Arrays^2^ which systematically profile lncRNAs together with protein-coding mRNAs. A typical workflow of transcriptomic analysis involves several steps: (1) sample preparation; (2) RNA isolation by TRIzol or other commercial kits; (3) high-quality RNA submitted to Core facility or commercial companies for RNA-seq; or reverse transcription to cDNA for hybridization-based microarray analysis (**Figure 1**). To visualize the result of data analysis, gene expression values from both transcriptomic analyses can be represented as heat maps, listing the most significantly changed genes in assays. Downstream analysis of microarray and RNA-seq are quite similar, include gene ontology (GO) enrichment and pathway analysis as well as functionally classification of gene annotation (Yue and Reisdorf, 2005).

## Advantages and Limitations of Transcriptome Profiling Technologies

Currently, microarrays remain a widely used approach for transcriptome studies due to its relatively low cost (readily affordable by many researchers) and ease to process large numbers of samples (Zhao et al., 2014). However, microarray has several limitations, most of which arise from probe and hybridization-related issues (probe performance and non-specific hybridization etc), such as high background level, difficult to detect very lowly expressed transcripts, and novel transcripts as well as splice variants (Draghici et al., 2006; Zhao et al., 2014). In contrast, RNA-seq has obvious advantages in these aspects (Russo et al., 2003; Wang et al., 2009; Zhao et al., 2014; Zhang et al., 2015) (**Table 1**): (1) Ability to detect novel transcripts; (2) Wider dynamic range of detection; (3) High signal-to-noise ratio; (4) High reproducibility and low variation. However, performing RNA-seq-based experiments is more expensive than microarray-based experiments, and requires extensive technical and bioinformatic expertise in data analysis (Zhao et al., 2014). The cost issue would potentially limit its application in experimental and clinical medicine. Moreover, a typical RNA-seq data in various formats was at the scale of GB depending on the number of samples tested. This presents a potential challenge for RAW data storage, processing, and analysis (Draghici et al., 2006). Fortunately, with recent technological advances, the costs for performing sequencing have declined; thus, RNA-seq is becoming more affordable than usual to users. Also various data depositing platforms (such as Gene Expression Ominbus and ArrayExpress) have emerged, and these platforms significantly solved the storage issue of large-scale RNA-seq RAW data. Readers are referred to references (Russo et al., 2003; Draghici et al., 2006; Wang et al., 2009; Zhao et al., 2014; Zhang et al., 2015) for details of the advantages and limitations of RNA-seq and microarray technology.

**Table 1:** Comparisons of qPCR array, microarray and RNA-sequencing.

Technology	Advantages	Limitations
qPCR Array	Low-cost; simple	Only testing limited number of genes of interest in specific pathways
Microarray	Low-cost; ability to process large number of samples; high-throughput	Low sensitivity for very lowly-or very highly expressed genes; high background; difficult to detect novel transcripts
RNA-seq	High accuracy; high sensitivity and dynamic range; low background/noise signal; high-throughput; identify novel transcripts, splice junctions, SNPs and non-coding RNAs	High-cost; high data storage

*SNPs, single nucleotide polymorphism*.

## Database Search

Traditionally, transcriptomic data were included as supplementary information in published scientific literature. Nowadays, to meet the need of open data and data sharing, most of the transcriptomic profiling data were deposited in ArrayExpress^3^ and NCBI Gene Expression Ominbus (GEO)^4^. In this study, I will summarize part of the datasets that has been deposited in GEO database with supported publication records.

## Basic Bioinformatics Analysis of Published Datasets

Currently, there are many softwares or websites that can help researchers analyze the data obtained from microarray and RNA-seq when uploading gene ID list. I summarize here some of the softwares and websites in **Table 2**. Basic bioinformatics analysis of transcriptomic data include the following (Yue and Reisdorf, 2005):

**Table 2:** Basic bioinformatic tools for gene profiling studies.

Downstream analysis	Tool software or website
GO analysis	Enrichr: http://amp.pharm.mssm.edu/Enrichr/ (Chen et al., 2013)
	Gene Ontology Consortium: http://www.geneontology.org/ (Ashburner et al., 2000)
	BiNGO: https://www.psb.ugent.be/cbd/papers/BiNGO/Home.html (Maere et al., 2005)
Pathway analysis	Enrichr: http://amp.pharm.mssm.edu/Enrichr/ (Chen et al., 2013)
	Qiagen Ingenuity pathway analysis: https://www.qiagenbioinformatics.com/products/ingenuity-pathway-analysis/
Venn Diagram	Gene Venn: http://genevenn.sourceforge.net/
	BioVenn: http://www.biovenn.nl/ (Hulsen et al., 2008)

*GO, gene ontology*.

(1) Gene Ontology (GO) analysis. One of the main uses of the GO is to perform enrichment analysis of target gene sets. For example, given a set of genes that are up-regulated under certain conditions, an enrichment analysis will find which GO terms are over-represented (or under-represented) using annotations for that gene set. There are mainly three types of GO analysis, i.e., biological process, molecular function and cellular component.
(2) Pathway analysis: After transcriptomic studies, we may find many genes that are differentially expressed under certain conditions. To summarize the specific pathways that mediated by those genes, two most commonly used pathway analysis-PANTHER and KEGG2016 are frequently used.
(3) Venn diagram analysis of overlapping genes. If multiple RNA-seq or gene arrays were performed, finding the overlapping genes can be quickly achieved by using the venn diagram to show common genes (for example, overlapping genes upregulated by vector-based overexpression or pharmacological agonists, but downregulated by siRNA treatment or pharmacological inhibitor treatment). This would help define a common transcriptional program directed by target gene or therapeutic intervention.

## Mining Transcriptomic Profiling Data

Due to the fact that gene microarray or RNA-seq generates big data that cannot be presented in regular format, therefore, it is critical to mine the data/information deposited in publicly available databases and perform related analysis.

### Analytical Steps

(1) Enter GEO accession number in GEO database
(2) Click “analyze with GEO2R,” which allows users to identify differentially expressed genes across various experimental conditions.
(3) Define sample groups and assign all samples (technical/biological replicates) in each group to minimize technical variation and improve reproducibility as instructed^5^.
(4) Perform the test and analyze top 250 differentially expressed genes or all whole gene sets. Adjusted *p*-value (after multiple-test correction) and log FC (fold change) are two important parameters for mining the data.

### An Example to Mine Gene Profiling Data

**FIGURE 2:**
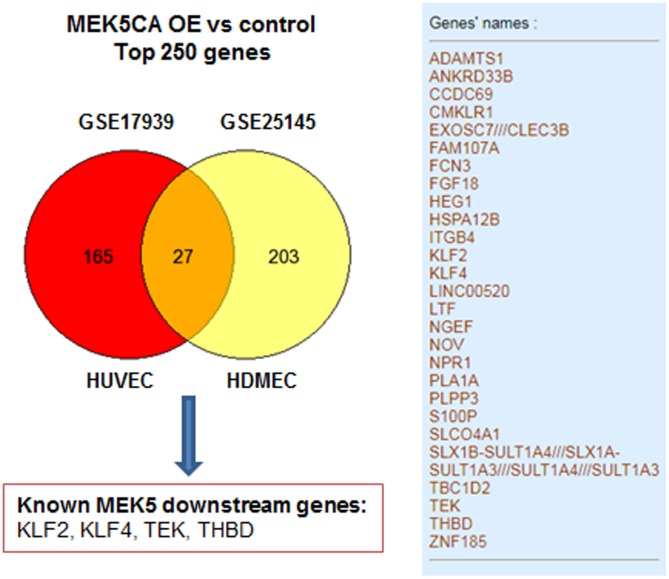
An example of mining existing data from public repository GSE17939 (Ohnesorge et al., 2010) and GSE25145 (Clark et al., 2011). A full list of 27 common genes revealed by both datasets was presented in the right panel. Total number of genes was less than 250 which is due to missing annotations in both datasets. MEK5CA OE, overexpression of constitutively active MEK5 mutant; HUVEC, human umbilical vein endothelial cells; HEMEC, Human dermal microvascular endothelial cells.

To exemplify the utility of data-mining, two transcriptomic studies GSE17939 (Ohnesorge et al., 2010) and GSE25145 (Clark et al., 2011), utilizing retrovirus-mediated overexpression of constitutively active mutant of MEK5 (MEK5-CA) in human primary endothelial cells was compared to study the transcriptiome of ERK5 activation. For simplicity, top 250 differentially expressed gene signature were mapped using venn diagram. As can be seen from **Figure 2**, transduction with MEK5-CA retrovirus, significantly altered 27 common genes in human umbilical vein endothelial cells and human dermal microvascular endothelial cells. Among the 27 genes, well-known downstream genes ensuing ERK5 activation, such as KLF2, KLF4, THBD, and TEK were identified. Venn diagram analysis also showed that MEK5-CA overexpression upregulates novel transcripts such as PLA1A and LINC00520, indicating both transcripts are potential MEK5 downstream effectors which may regulate endothelial function.

## Transcriptomic Profiling in Cell, Animal Experiments and Human Patients

Transcriptomic comparisons would facilitate the identification of differentially expressed transcripts between human diseased and control samples, in different vascular cell types (endothelial cells, monocytes/macrophages, and smooth muscle cells), or in response to different pharmacological/genetic/environmental perturbations (Musunuru et al., 2017). Three common types of transcriptome profiling in vascular biology are summarized as below (**Tables 3**–**5**):

**Table 3:** Gene profiling studies of vascular diseases in human patients.

Sample comparison	GEO accession#	Reference
Carotid atheroma vs. adjacent plaque-free carotids	GDS5083	Ayari and Bricca, 2013
Abdominal aorta aneurysms vs. abdominal aorta control	GDS2838	Hinterseher et al., 2011
Abdominal aorta aneurysms vs. abdominal aorta control	GSE7084	Lenk et al., 2007
Ruptured vs. stabilized plaques	GSE41571	Lee et al., 2013
Early vs. advanced atherosclerotic plaques	GSE28829	Doring et al., 2012
Peripheral blood from female atherosclerotic vs. non-atherosclerotic patients	GSE20129	Liu et al., 2016
Platelets from CAD patient and healthy control	GSE59421	Kok et al., 2015

*CAD, coronary artery disease*.

**Table 4:** Gene profiling studies of vascular diseases in experimental animal models.

Sample comparison	GEO accession#	Reference
Diabetic ApoE^-/-^ mice vs. control ApoE^-/-^ mice	GDS3755	Bu et al., 2010
ApoE^-/-^ mice + HFD vs. ApoE^-/-^ mice + ND	GSE83112	Bao et al., 2016
Vitamin E-treated ApoE^-/-^ mice vs. vehicle treatment	GSE42813	Abd Alla et al., 2013
ApoE^∗^3 Leiden mice treated with rosuvastatin and ezetimibe vs. vehicle	GSE38688	Verschuren et al., 2012
ApoE^-/-^ mice treated with captopril vs. vehicle	GDS3683	Abd Alla et al., 2010
ApoE^-/-^ mice treated with rosiglitazone vs. vehicle	GSE28031	Abd Alla et al., 2016
Ang-II induced AAA in ApoE^-/-^ mice vs. saline control	GSE17901	Spin et al., 2011
Ang-II induced AAA in ApoE^-/-^ aorta vs. AAA-resistant aorta and control aorta	GSE12591	Rush et al., 2009
Elastase-induced AAA C57BL/6J mice aorta vs. control	GSE51228	Maegdefessel et al., 2014
Atherosclerosis prone vs. resistant regions of ApoE^-/-^ aorta	GSE13836	Van Assche et al., 2011

*AAA, abdominal aorta aneurysms; Ang-II, angiotensin II; HFD, high fat-diet; ND; normal chow-diet*.

**Table 5:** Gene profiling studies in cultured vascular cells.

Cell type	Treatment	GEO accession#	Reference
Endothelial Cells	Different degree of laminar shear stress	GSE23289	White et al., 2011
	Pulsatile, oscillatory shear stress	GSE92506	Huang et al., 2017
	Laminar shear stress	GSE71164	Maleszewska et al., 2016
	Laminar shear stress in young and senescent cells	GSE13712	Mun et al., 2009
	Low shear stress, high shear stress, reversing flow	GSE16706	Conway et al., 2010
	MEK5/CA	GSE17939 GSE25145	Ohnesorge et al., 2010; Clark et al., 2011
	Ox-PAPC, TNFα, and IL1β	GSE72633	Briot et al., 2015
	Acrolein	GSE56782	O’Toole et al., 2014
	IL4	GSE28117	Tozawa et al., 2011
	oxLDL	GDS4262	Mattaliano et al., 2009
	HDL	GSE53315	Tabet et al., 2014
	Atorvastatin	GSE2450 GSE8686	Boerma et al., 2006, 2008
	High glucose	GSE30780	Pirola et al., 2011
Vascular Smooth Muscle Cells	Ang II	GSE38056	Leung et al., 2013
	Homocysteine	GDS3413	Van Campenhout et al., 2009
	Nebivolol or metoprolol	GDS2021	Wolf et al., 2008
	Atg7-SMC-KO	GSE54019	Grootaert et al., 2015
	IL1	GSE21403	Alexander et al., 2012
	oxLDL	GSE36487	Minta et al., 2010
	2-methoxyestradiol	GSE12261	Rigassi et al., 2015
	Fluid shear stress	GSE19909	Ekstrand et al., 2010
Macrophages	oxLDL	GSE54039 GSE32358 GSE54975 GSE58913	Gold et al., 2012; Hu et al., 2014; Ramsey et al., 2014; Reschen et al., 2015
	Ac-LDL	GSE24894	Kim et al., 2011
	HDL	GSE44034	De Nardo et al., 2014
	LPS	GSE32359	Gold et al., 2012
	CXCL4	GDS3787	Gleissner et al., 2010
	Palmitate	GSE98303	Oteng et al., 2017
	IFNγ and LPS (M1), IL-4 (M2a), IL10 (M2c)	GSE57614	Derlindati et al., 2015
	Hypochlorous acid	GSE15457	Woods et al., 2009
	Simvastatin	GSE4883	Tuomisto et al., 2008
	GW3965	GSE70444	Ito et al., 2015
	STX4	GSE39079	Feldmann et al., 2013
	Anti-miR-33	GSE28783	Rayner et al., 2011

*MEK5/CA, MEK5 constitutively active mutant; IL, interleukin; oxLDL; oxidized LDL; Ac-LDL, acetylated LDL; HDL, high-density lipoprotein; ox-PAPC, oxidation product of 1-palmitoyl-2-arachidonyl-sn- glycero-3-phosphorylcholine; CXCL4, chemokine (C-X-C motif) ligand 4*.

### Human Diseased Samples vs. Controls

Comparing different expression profiles of genes in normal (disease-free, or mild disease, or adjacent non-disease regions) and pathological tissues in the majority of cases can represent both a cause and a consequence of the disease. Given the fate of atherosclerotic plaques can be divided into stabilized (asymptotic) and vulnerable plaques (symptomic), and most acute cardiovascular events are caused by the rupture of vulnerable plaques (Jackson, 2011), thus mining of these data will yield valuable information regarding key genes that regulate plaque stability.

### Mice Diseased Samples vs. Controls as well as Cardiovascular Drugs Treatment vs. Control Treatment

Compared with human samples with vascular diseases, mice samples are easier to be obtained by diet/chemical induction. In this regard, ApoE^-/-^ and LDLr^-/-^ mice were two of the most frequently used mouse strains for transcriptomic analysis in vascular diseases, atherosclerosis and abdominal aorta aneurysms (AAAs) in particular (Emini Veseli et al., 2017). In addition, mice are very useful in evaluating vasculoprotective drugs. Comparing differential gene expression among aortas from hyperlipidemic mice treated with drugs or vehicle control could yield important mechanistic insights into drugs’ vasculoprotective actions and mechanisms.

Since hyperlipidemia represents a key risk factor that drives multiple cardiometabolic diseases including atherosclerosis, Novák et al. (2015) have recently reviewed miRNAs in cholesterol, fatty acid metabolism and atherosclerosis. This review highlights the complexity and importance of gene regulation by miRNA in the context of vascular diseases. However, the quest for disease-associated miRNA and target genes has been hampered by research tools, and fortunately, this difficulty can be tackled by computational prediction, followed by target validation (Lagana, 2015).

### Gene Profiling Studies in Vascular Cells

Endothelial dysfunction is the underlying cause for multiple cardiometabolic diseases (Fang et al., 2017). However, endothelial dysfunction can be prevented by lipid-lowering statins, laminar flow, as well as naturally occuring phytochemicals. For example, recently, Maleszewska et al. (2016) has conducted RNA-seq analysis to interrogate the transcriptome of endothelial cells in response to fluid shear stress. This deep transcriptomic analysis of endothelial cells in the context of atheroprotective shear stress, together with other recently published transcriptomic profiling data (Qiao et al., 2016), constitute useful resources to further explore functions of mechanosensitive genes and lncRNAs in endothelial cell biology. There are also many deposited GEO datasets evaluating the effects of disease-associated stimuli (such as angiotensin II and oxidized LDL)/disease-modifying drugs (such as statins) on smooth muscle cells function (proliferation and migration) and macrophage function (inflammation, lipid loading, and polarization). In the GEO database, mining of these data will advance our understanding of the patho-mechanisms of atherosclerosis, which would potentially lead to novel anti-atherosclerotic therapy.

## Discussion and Conclusion

Genome-wide transcriptomic analysis by microarray and RNA-seq emerge as powerful tools for translational research. Serval studies have conducted microarray and RNA-seq in parallel to compare the similarity and difference in transcriptome profiling of target cells/tissues and found that there is a high concordance between two technologies, suggesting the general suitability and reliability of both technologies (Bottomly et al., 2011; Sirbu et al., 2012; Zhao et al., 2014). Both technologies greatly transformed our traditional method of research into “discovery”-based method for mechanistic studies, allowing us to readily evaluate the cell-type and stimulus (or small-molecule drug)-specific regulation of gene expression. From these studies, readers can also mine data according to web-based instructions, and develop a working hypothesis to test whether gene X is involved in the development and progression of vascular diseases. Due to the complex nature of transcriptional regulation, the level of specific transcripts is determined by both transcriptional machinery and environment (such as diet, nutrient etc) (Musunuru et al., 2017). Together with recently emergence of single cell RNA-seq (Linnarsson and Teichmann, 2016), these biotechnological advances will provide powerful toolboxes for understanding the vascular transcriptome and represent an import step toward precision cardiovascular medicine.

Overall, RNA-seq is a high-throughput, and “discovery-based” approach for investigating transcriptome of different samples. It is more sensitive in detecting very lowly expressed genes or extremely highly expressed genes, therefore, offers a wider dynamic range than traditional microarray (Zhao et al., 2014). It is replacing microarray and thus becoming the predominant tool for transcriptome studies in basic, translational and clinical research (Musunuru et al., 2017). Noteworthy, RNA-seq is also a powerful tool for investigating non-coding RNA, lncRNAs in particular [such as SMILR (Ballantyne et al., 2016), MANTIS (Leisegang et al., 2017), LincRNA-p21 (Wu et al., 2014), Lnc-Ang362 (Leung et al., 2013), MYOSLID (Zhao et al., 2016), RNCR3 (Shan et al., 2016)], regulates many facets of vascular biology (reviewed in Li et al., 2016; Poller et al., 2017), demystifying the “dark” genome of vascular diseases. These lncRNA emerge as important players and therapeutic targets in vascular diseases. However, we have to bear in mind that: different variables of experimental conditions (such as cell types, treatment time, and animal models) pose a challenge to make quantifiable conclusions from published datasets. At least, by mining and comparing different datasets from different studies, we can gain a general comprehension on specific genes that are commonly regulated by the same treatment/disease.

The invention of new datamining tools/softwares is a good strategy to mine existing data. Recently, a web-based tool, Transcriptomine (Becnel et al., 2017), was developed to mine data on dissecting the effects of genetic or pharmacological interventions on nuclear receptor signaling. We can envisage that deep mining of the “hiden” data in vascular medicine will definitely accelerate biomarker discovery and prompts the identification and functional characterization of novel therapeutic targets (including coding genes and lncRNAs) in vascular diseases including atherosclerosis, AAA, and other rare-occuring vascular diseases. To conclude, mining expression profiling data from bench to bedside represents a cost-efficient new avenue for research of precision cardiovascular medicine.

## Author Contributions

SX contributed to the conception, drafting, and revision of the manuscript and approved the final version.

## Conflict of Interest Statement

The author declares that the research was conducted in the absence of any commercial or financial relationships that could be construed as a potential conflict of interest.

## Footnotes

1 https://professional.heart.org

2 http://www.arraystar.com/lncrna-array-service/

3 https://www.ebi.ac.uk/arrayexpress/

4 https://www.ncbi.nlm.nih.gov/geo/

5 https://www.ncbi.nlm.nih.gov/geo/info/geo2r.html
